# Impact Damage Detection Using Chirp Ultrasonic Guided Waves for Development of Health Monitoring System for CFRP Mobility Structures

**DOI:** 10.3390/s22030789

**Published:** 2022-01-20

**Authors:** Langxing Tan, Osamu Saito, Fengming Yu, Yoji Okabe, Taku Kondoh, Shota Tezuka, Akihiro Chiba

**Affiliations:** 1 Institute of Industrial Science, The University of Tokyo, 4-6-1 Komaba, Meguro-ku, Tokyo 153-8505, Japan; osaito@iis.u-tokyo.ac.jp (O.S.); houmei@iis.u-tokyo.ac.jp (F.Y.); 2 Yamaha Motor Company Limited, 2500 Shingai, Iwata, Shizuoka 438-8501, Japan; kondoutaku@yamaha-motor.co.jp (T.K.); tezukas@yamaha-motor.co.jp (S.T.); chibaa@yamaha-motor.co.jp (A.C.)

**Keywords:** structural health monitoring, carbon fiber-reinforced plastic (CFRP), impact damage, chirp signal, Lamb waves

## Abstract

When impact damage occurs in carbon fiber-reinforced plastic (CFRP) structures, it is barely visible but may cause significant degradation in the mechanical properties of the structure. Hence, a structural health monitoring (SHM) system that can be installed in CFRP mobility structures and is sensitive to impact damage is needed. In this study, we attempted to establish an SHM system based on ultrasonic guided waves, which are generated by inputting a broadband chirp signal into a film-like piezoelectric actuator. The relationship between impact damage size and maximum time-of-flight (ToF) delay was investigated for three types of CFRP plates: woven, non-woven, and hybrid laminates. As a result, it was found that the maximum ToF delay increased linearly with an increase in the damage size for all CFRP laminates. Moreover, the amplitude of the A_0_ mode was found to be significantly affected by the damage length in the wave propagation direction. Thus, this SHM method using chirp ultrasonic waves can quantitatively evaluate the size and extent of the impact damage in CFRP laminates.

## 1. Introduction

Presently, various carbon fiber-reinforced plastic (CFRP) laminates are applied to personal mobility structures because of their lightweight and high-strength properties. However, personal mobility structures made of CFRP laminates are likely to suffer from barely visible impact damages (BVIDs) of which the owner may be unaware [1]. BVIDs can cause significant degradation in performance and seriously affect the safety of daily use [2]. Hence, structural health monitoring (SHM) systems that can detect BVIDs in CFRP laminates are expected to be installed into the structures [3].

Lamb waves are promising and widely applied in the construction of SHM systems. Because Lamb waves can propagate for long distances with small attenuation [4,5], a large area can be monitored from a single actuator. Furthermore, Lamb waves can examine various types of flaws in different materials, including cracks, fatigue-based defects, debonding, and impact damage. Thus, many studies have been conducted on SHM based on Lamb waves [6]. For example, Yu et al. quantified the crack damage in an aluminum plate using guided-wavefield analysis methods [7]. Qiu et al. validated the propagation of fatigue cracks in real aircraft structures under realistic loads based on a guided wave-Gaussian mixture model [8]. Okabe et al. evaluated the debonding progress in the CFRP skin/stringer structural element of airplanes [9].

To detect impact damage in CFRP structures, we can use the velocity change of the lowest antisymmetric Lamb wave (A_0_) mode [10,11]. When impact damage occurs in a CFRP laminate, the velocity of the A_0_ mode propagating through the damaged area is reduced because of the stiffness reduction. Hence, by observing the time of flight (ToF) of the A_0_ mode, we can detect the impact damage. 

In general, ToF is obtained using tone burst waves as input signals to excite Lamb waves [12,13]. One of the advantages of the tone burst signal is that the undesirable Lamb wave mode will not be generated because the frequency bandwidth is limited [14]. However, tone burst signals are not effective in evaluating impact damage in CFRPs because the interaction between Lamb waves and impact damage is intricate and the optimal frequency will be different for different impact damages. Thus, to reliably detect the impact damage, we need to observe ToF over a broad bandwidth. Although it is possible to repeat tone burst excitations by changing the central frequency, the task is inconvenient and time-consuming. By contrast, using a broadband signal to excite Lamb waves is more efficient for detecting and evaluating impact damage comprehensively.

In this study, we attempted to evaluate the impact damage in CFRP laminates based on broadband chirp signal excitation. In a chirp signal, the frequency changes as a function of time. By using chirp signals, the measurement time can be reduced and a high signal-to-noise ratio can be achieved [15]. However, the analysis of the received signal is difficult because of the multimode excitation and dispersive behavior of Lamb waves. To simplify the analysis, we used a transfer function that relates the input signal and the response signal in the frequency domain [16]. By using the transfer function obtained with the chirp signal, we can calculate the response signals of the tone burst excitation with certain central frequencies. Thus, ToF can be evaluated comprehensively at various frequencies. Accordingly, our proposed method is efficient in data acquisition and reliable for impact damage detection.

We used macro-fiber composites (MFCs) as transducers to excite and receive Lamb waves. MFCs have been widely applied to SHM systems since they were developed by NASA in 1999 [17,18]. An MFC is a film-like device that consists of aligned rectangular piezoceramic fibers sandwiched between epoxy layers and polyimide films with electrode pattern. Through the attached electrodes, voltage signals are transferred to the inside piezoceramic fibers [19,20]. MFCs have broadband characteristics, which are important for our purpose. In addition, MFCs have many advantages such as their light weight, high flexibility, ability to work in severe conditions, and low price. Hence, MFCs are suitable for SHM systems installed in mobility structures.

The remainder of this paper is organized as follows. We begin by observing the cross-section of the impact-damaged area in all three types of CFRP plates to clarify the damage conditions of the laminates in Section 2. In Section 3, the data processing method using a transfer function is discussed. In Section 4, our experimental investigation of the relationship between the impact damage size and the ToF delay in various CFRP plates is presented. We also investigated the influence of the shape of the impact damage on the waveform and clarified the change in the amplitude of the A_0_ mode when the damage was enlarged in the direction of wave propagation. Section 5 concludes the paper.

## 2. Cross-Sectional Observation of Impact-Damaged Area

Various CFRP plates with different layup configurations are used in manufacturing mobility structures. From the viewpoint of practical applications, the SHM system should be able to detect impact damage in various CFRP plates. Hence, three types of CFRP plates were used in this study: woven CFRP plates ([(0, 90)/(45, −45)]_4s_, 3.64 mm in thickness), non-woven CFRP plates ([0/90/45/−45/−45/45/90/0]_s_, 3.84 mm in thickness), and hybrid CFRP plates ([(0, 90)/(45, −45)/0/90/(0, 90)/(45, −45)/(0, 90)/(45, −45)]_s_, 3.7 mm in thickness). The hybrid CFRP plate was laminated using both woven and non-woven prepreg sheets. 

Because the layup configurations are different in these CFRP plates, the microscopic damage inside the impact-damaged area may also be different for different plates. For a better diagnosis of impact damage, it is important to know the microscopic damage information in each plate after impact events. Hence, in this section, impact damage is formed in the laminates, and the cross sections of the impact-damaged areas are observed in the woven, non-woven, and hybrid CFRP plates.

An impact load was applied to the plates using a drop-weight impact tester with an impact energy of 15.9 J. The diameter of the impactor was 12.5 mm. The impact damages in all the CFRP laminates were barely visible from the outside, even after the application of a 15.9 J impact load. Then the specimens were cut through the center of the impacted area, and the cross sections of the impact-damaged area were observed using a digital microscope (VHX-950F, Keyence Corp., Osaka, Japan).

**Figure 1:**
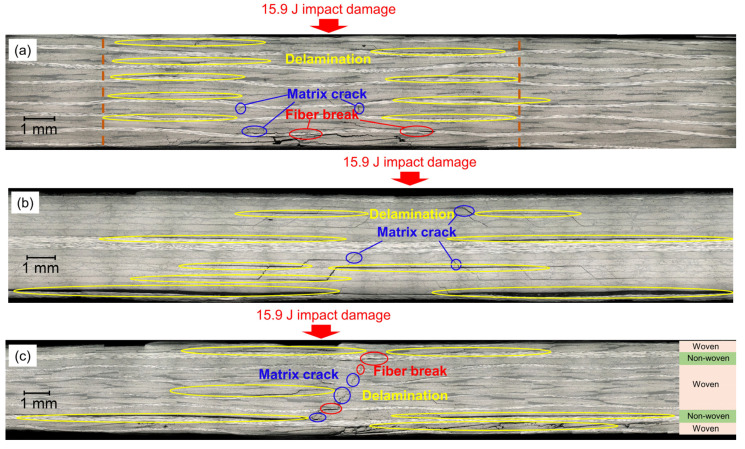
Cross-sectional observation of the impact-damaged area in (**a**) the woven CFRP plate, (**b**) the non-woven CFRP plate, and (**c**) the hybrid CFRP plate. Different microscopic damages are denoted using circles with different color.

Figure 1a shows the cross-section of the woven CFRP plate. There are three main types of microscopic damage in the impact damage area: delamination, matrix cracks, and fiber breaks. Because delaminations are distributed evenly over the entire damaged area, the impact damage can be assumed to be an area with a uniform stiffness degradation [11]. The diameter of the damaged area is almost constant throughout the thickness, as shown by the orange dashed lines, which is about 15 mm. 

Next, we show the observation results of the non-woven CFRP plate in Figure 1b. There was no fiber breakage, and delamination was found to be the most dominant microscopic damage. In particular, the delaminations at the interface nearest to the bottom surface are the largest, whose diameter is about 25 mm. The distribution of the delamination is not even in the non-woven CFRP plate; this is contrary to the woven CFRP plate shown in Figure 1a.

Cross-sectional observations of the hybrid CFRP plate are shown in Figure 1c. Delaminations mainly exist at the interfaces between the non-woven plies and woven plies. Similar to the non-woven CFRP plate, the largest delamination appeared at the lower interfaces, whose diameter is also about 25 mm. Matrix cracks and fiber breaks were also observed in the woven plies. Thus, the microscopic damage in the hybrid CFRP plate is complicated and can be regarded as a mixture of the damage in the woven and non-woven CFRP plates.

Although the impact damages are barely visible from the outside in these CFRP laminates, the internal damage is severe and the damage conditions differ depending on the layup configuration. A practical SHM system must detect the impact damage in various CFRP plates, even if the internal microscopic damages are different. 

## 3. Data Collection and Data Processing Method

### 3.1. Experimental Procedure

**Figure 2:**
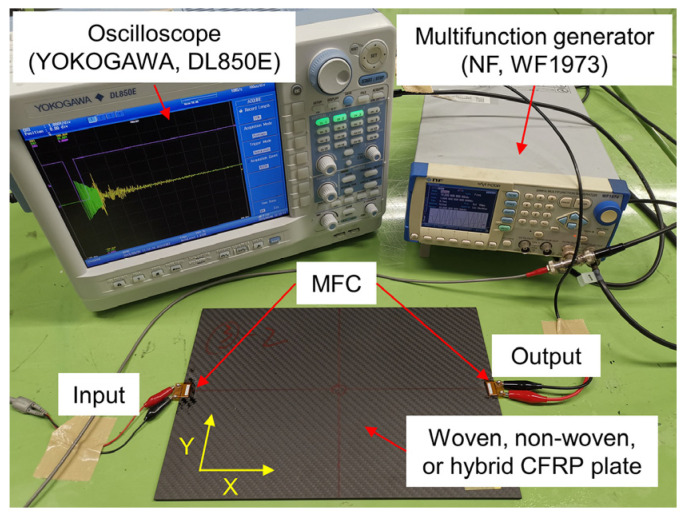
Experiment setup for detection of impact damage in a CFRP plate with MFC transducers.

Experiments to detect impact damage were conducted on CFRP plates. The experimental setup is shown in Figure 2. Three types of CFRP plates with dimensions of 300 × 205 mm^2^ were used in the experiments.

MFCs were used as actuators and sensors to excite and receive guided waves. The size of the MFC transducers (M-0714-P2, Smart Material Corp, Sarasota, FL, USA) used in this study were 7 mm in length and 14 mm in width. One of the MFCs was bonded at the middle of the left edge of the CFRP plate and used as the actuator to convert the electrical signal into the strain to excite ultrasonic waves. Meanwhile, another MFC was bonded to the other side of the plate as the receiver. The distance between the two MFCs was 300 mm. A multifunction generator (WF1973, NF Corp., Kanagawa, Japan) was used to generate electrical input signals. 

A chirped signal was chosen as the input signal because of its broadband characteristic. A typical chirped signal with a constant amplitude is expressed by Equation (1) [15]

(1) $$s_{c}\left(t\right)=w\left(t\right)\sin\left(2\pi f_{0}t+\frac{\pi Bt^{2}}{T}\right)$$

in Equation (1), $f_{0}$ is the minimum starting frequency, B is the chirp bandwidth, T is the duration of the chirp signal, and w(t) is a unit amplitude rectangular window starting at t=0 with a duration of T. The frequency increases linearly from $f_{0}$ to $f_{0}+B$ during time duration T.

**Figure 3:**
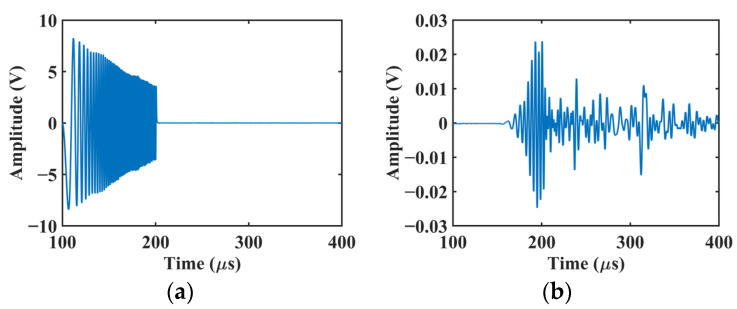
Temporal waveforms of (**a**) an input chirp signal and (**b**) a response signal.

The parameters of the chirp input signal to the MFC were set to be $f_{0}=10$ kHz, B=990 kHz, and T=100 μs, and the amplitude was 8.5 V. Figure 3a shows the input waveform recorded with a digital oscilloscope (DL850E, Yokogawa Test & Measurement Corp., Tokyo, Japan). In the actual vibration, the amplitude decreased with an increase in the frequency due to the impedance mismatch between the MFC and the multifunction generator. Figure 3b shows the output waveform obtained after 4096-time averaging for noise reduction.

### 3.2. Transfer Function Method

Although the data acquisition is efficient in the case of chirp signal excitation, the direct analysis of the received signal is difficult because many Lamb wave propagation modes can be excited and they are dispersive [5]. Therefore, we extracted signals in response to tone bursts at certain frequencies from the signals in response to the broadband chirp input. To this end, we introduced a transfer-function method.

The ultrasonic wave propagation system, which consists of an actuator, a target structure, and a receiver, can be modeled as a linear time invariant (LTI) system [15]. For a linear ultrasonic propagation system, the relation between the input signals s(t) and the response signal r(t) can be expressed in the frequency domain as:

(2) R(ω)=H(ω)S(ω),

where S(ω) is the Fourier transform of the input signal s(t), R(ω) is the Fourier transform of the response signal r(t), and H(ω) is the system’s transfer function. The system’s transfer function H(ω) includes Green’s function to represent the wave propagation, and the effects of all the instrumentation, such as the actuators and sensors in the system [15]. 

The transfer function H(ω) over a broad bandwidth can be determined experimentally by the excitation and reception of a chirp ultrasonic wave as:

(3) $$H\left(\omega \right)=\frac{R_{c}\left(\omega \right)}{S_{c}\left(\omega \right)}\;,$$

where $S_{c}\left(\omega \right)$ is the Fourier transform of the input chirp signal $s_{c}\left(t\right)$ (Figure 3a), and $R_{c}\left(\omega \right)$ is the Fourier transform of the response signal $r_{c}\left(t\right)$ (Figure 3b). 

After obtaining the transfer function over a broad bandwidth, the response signal for any known tone burst whose frequency falls within the range of the transfer function bandwidth, can be calculated. Let $s_{b}\left(t\right)$ represent the tone burst excitation in the time domain and $S_{b}\left(\omega \right)$ be its Fourier transform. The response signal $R_{b}\left(\omega \right)$ can be calculated as:

(4) $$R_{b}\left(\omega \right)=H\left(\omega \right)S_{b}\left(\omega \right).$$

Then, the response signals in the time domain $r_{b}\left(t\right)$ are calculated by the inverse Fourier transform from $R_{b}\left(\omega \right)$. 

**Figure 4:**
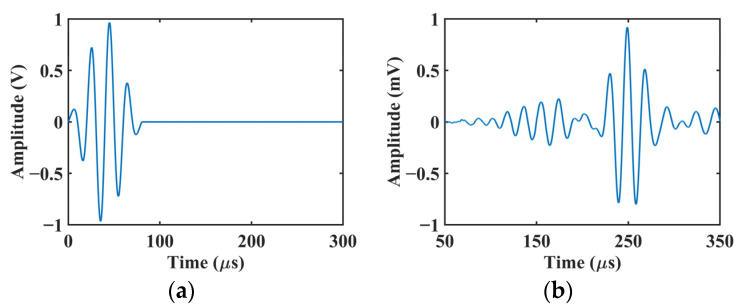
Temporal waveforms of (**a**) numerical input tone burst signal and (**b**) reconstructed response signal.

As an illustration, we consider 4-cycle sinusoidal tone burst signals at 50 kHz with a Hamming window as the numerical input signal. The input signal and reconstructed response signal are shown in Figure 4a,b, respectively. Thus, this method can be applied to reconstruct the response signals from known tone burst input signals at certain frequencies within the frequency range of the input chirp.

The transfer function varies if impact damage occurs in the structure. The change in the transfer function of the system can be determined similarly by a subsequent experiment using chirp ultrasonic excitation. Similar to Equation (3), the changed transfer function can be expressed as:

(5) $$H^{\prime }\left(\omega \right)=\frac{R_{c}^{\prime }\left(\omega \right)}{S_{c}\left(\omega \right)}\;,$$

where $R_{c}^{\prime }\left(\omega \right)$ is the response signal in the frequency domain obtained experimentally after impact loading, and $H^{\prime }\left(\omega \right)$ is the transfer function changed by impact damage. The response signals $R_{b}^{\prime }\left(\omega \right)$ in the damaged condition can be calculated as:

(6) $$R_{b}^{\prime }\left(\omega \right)=H^{\prime }\left(\omega \right)S_{b}\left(\omega \right),$$

Then, response signals in the time domain $r_{b}^{\prime }\left(t\right)$ in the damaged condition are calculated by inverse Fourier transform from $R_{b}^{\prime }\left(\omega \right)$. By comparing the damaged signal $r_{b}^{\prime }\left(t\right)$ with the intact signal $r_{b}\left(t\right)$, we can detect the impact damage.

## 4. Damage Detection in Various CFRP Plates

### 4.1. Calculation of ToF Delay

**Figure 5:**
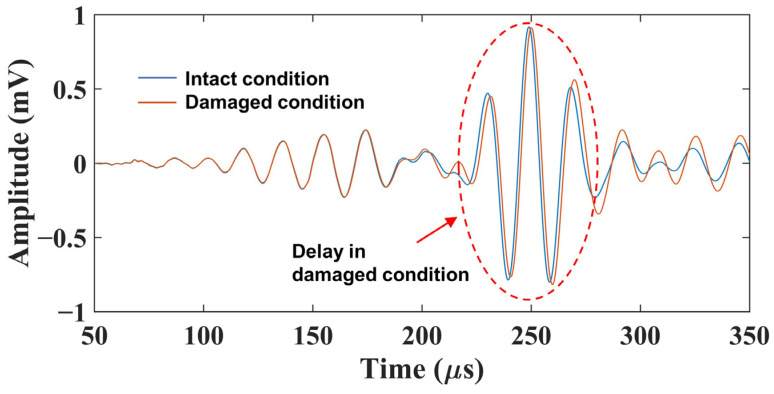
Comparison of reconstructed signals at 50 kHz between the intact and damaged conditions.

If there is impact damage in the propagation path, the group velocity of Lamb waves changes in the damaged area, leading to a change in the ToF of Lamb waves. The increase in the ToF in the damaged condition from that in the intact condition is called “ToF delay” in this article. By calculating the ToF delay, we attempted to detect the impact damage. In Figure 5, the reconstructed signals at 50 kHz in the intact and damaged conditions are compared. It can be seen that there is an increase in the ToF from the signal in the intact condition $r_{b}\left(t\right)$ to that in the damaged condition $r_{b}^{\prime }\left(t\right)$.

We calculated the ToF delay as follows: The $r_{b}^{\prime }\left(t\right)$ can be assumed to be a signal that is shifted from the $r_{b}\left(t\right)$ along the time axis while its waveform is maintained. Hence, shifting the $r_{b}^{\prime }\left(t\right),$ and finding the time difference corresponding to the best matching of the two signals, we can determine the ToF delay. To quantitatively evaluate the degree of matching, cross-correlation analysis is introduced. The cross-correlation coefficient $R_{c-c}$ as a function of the time difference $t_{d}$ is expressed as follows:

(7) $$R_{c-c}\left(t_{d}\right)=\underset{t}{\sum }r_{b}\left(t\right)r_{b}^{\prime }\left(t-t_{d}\right).$$

**Figure 6:**
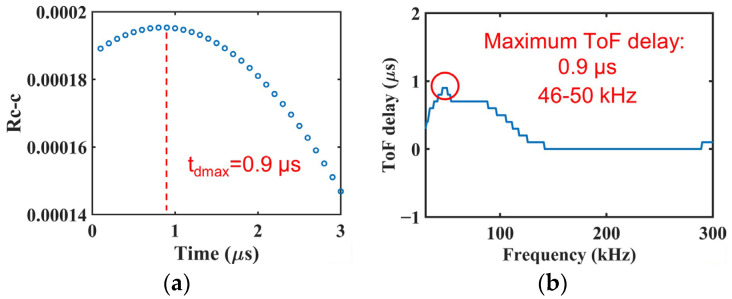
Results of calculating the ToF delay using cross-correlation analysis: (**a**) cross-correlation coefficient between the two signals as a function of time difference. (**b**) ToF delay distribution over a broad bandwidth after impact loading.

The $R_{c-c}\left(t_{d}\right)$, which indicates the degree of matching, increases as the two signals exhibit better matching and becomes the maximum value at the best matching. Hence, the time difference $t_{\mathrm{dmax}}$ corresponding to the maximum of $R_{c-c}$ is considered to be the ToF delay of the response signal $r_{b}^{\prime }\left(t\right)$. In Figure 6a, the cross-correlation coefficient $R_{c-c}$ at a frequency of 50 kHz in a woven CFRP plate is plotted against the time difference $t_{d}$. It can be seen that the time difference is 0.9 μs when the cross-correlation coefficient $R_{c-c}$ reaches the maximum value. As a result, the ToF delay was 0.9 μs.

Calculating the ToF delay at different frequencies, we plotted the ToF delay against the frequency, as shown in Figure 6b. The maximum ToF delay and corresponding frequency are easily recognized in this figure. This allows the maximum ToF delay of Lamb waves to be calculated using cross-correlation analysis.

### 4.2. Relation between Impact Damage Size and ToF Delay

In practical operation, impact damages with different sizes may occur in mobility structures. These damages need to be evaluated in the mobility structure SHM system. In this section, the relation between the impact damage size and ToF delay is investigated in the woven, non-woven, and hybrid CFRP plates.

**Figure 7:**
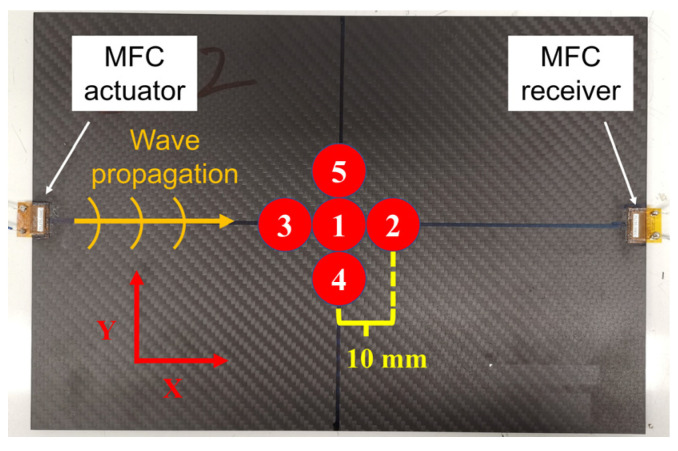
Positions of the five impacts.

After the chirp excitation experiment in the intact condition, the impact damages were given and enlarged by repeating the drop-weight impact test five times with a 12.5-mm impactor at 15.9 J. To avoid penetrating the CFRP plates with too much impact energy, the positions of the impact loading were changed gradually. As shown in Figure 7, the impact positions were 10 mm away from the center of the first impact damage. After the impact damage was created by the first impact, it was enlarged in the Lamb wave propagation direction (X direction) in the second and third impacts. Then, the damage was enlarged in the direction orthogonal to the propagation direction (Y direction) in the fourth and fifth impacts.

**Figure 8:**
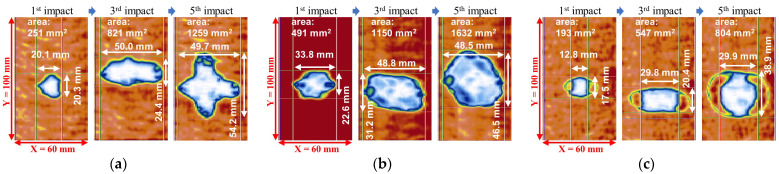
C-scan observation results of impact damages in (**a**) woven CFRP plate, (**b**) non-woven CFRP plate, and (**c**) hybrid CFRP plate.

After each impact loading, the damaged area was observed using an ultrasonic C-scan device (OMNISX-PA1664PR, OLYMPUS, Tokyo, Japan). C-scan images of the different plates are shown in Figure 8. At the time of the third impact, the length in the X direction increased significantly, while the length in the Y direction changed slightly. In contrast, at the time of the fifth impact, the length in the X direction was almost constant, and the damage was enlarged in the Y direction.

**Figure 9:**
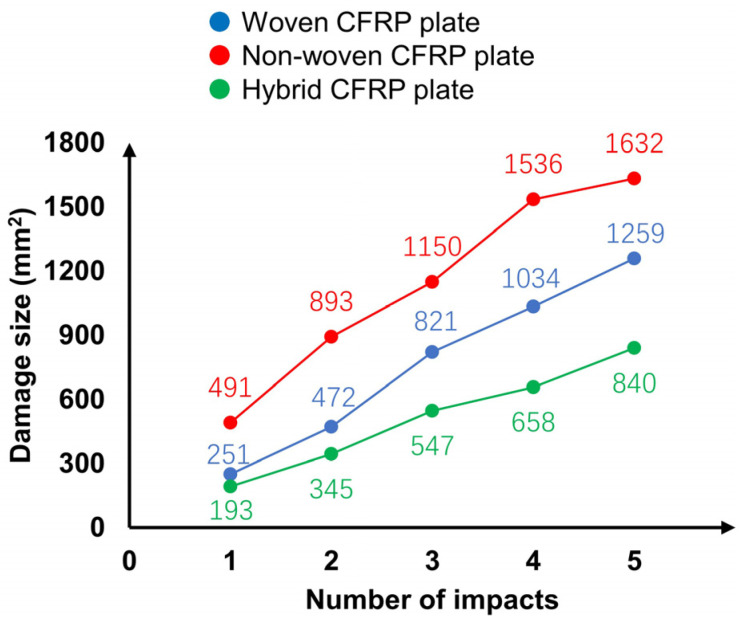
Changes in damage size with the number of impacts.

When estimating the size of the impact damage, a specific shape, such as an ellipse, cannot be assumed, because the shape of the impact damage changes after impact loading. Thus, the damage size was estimated by counting the pixels in the observation image as follows:

(8) $$S_{d}=\frac{N_{d}}{N_{r}}S_{r},$$

where $S_{d}$ is the estimated size of the damaged area, $N_{d}$ is the number of pixels in the damaged area, $N_{r}$ is the number of pixels inside the rectangle circumscribing the damaged area, and $S_{r}$ is the size of the circumscribing rectangle. The estimated size of the damaged area after impact loading in each plate is shown in Figure 9. 

After the drop-weight impact test, chirp ultrasonic propagation was conducted to obtain the transfer function in the increasingly damaged condition. The response signals to 4-cycle tone burst signals from 30 kHz to 300 kHz with 1-kHz intervals were reconstructed. Then, the ToF delay in each damaged condition was calculated using cross-correlation analysis for each plate over the above frequency range. Subsequently, the maximum ToF delay was obtained for each damaged condition. 

**Figure 10:**
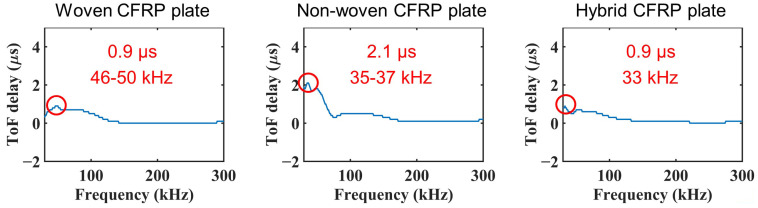
ToF delay after 15.9 J impact loading on each plate.

Figure 10 shows the ToF delay after the first impact loading in the woven, non-woven, and hybrid CFRP plates. The ToF delay clearly appeared for all plates. However, the distributions of the ToF delay in the frequency range are quite different for different plates. The maximum ToF delay appears at 46–50 kHz in the woven CFRP plate, 35–37 kHz in the non-woven CFRP plate, and at 33 kHz in the hybrid CFRP plate. Therefore, it is necessary to calculate the ToF delay over a broad bandwidth, because the frequency of the maximum ToF delay may vary depending on the different types of CFRP plates.

**Figure 11:**
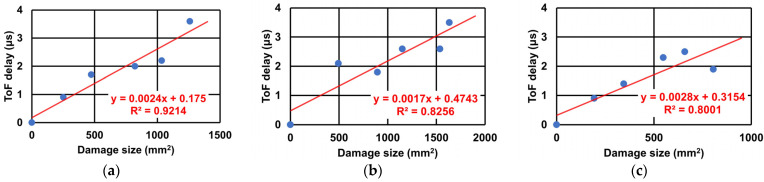
Relation between impact damage size and maximum ToF delay in (**a**) woven CFRP plate, (**b**) non-woven CFRP plate, and (**c**) hybrid CFRP plate.

The relationship between the size of the damage area and the maximum ToF delay in the three different CFRP plates is plotted in Figure 11. The result indicates that the ToF delay increases in proportion to the damage size in all three types of CFRP plates. The result of the woven CFRP (Figure 11a) shows the best linearity among the three plates. In non-woven CFRP (Figure 11b), the maximum ToF delay shows a growing trend as the damage size increases, although there are some slight fluctuations. For the hybrid CFRP plate (Figure 11c), the ToF delay decreased after the fifth impact. This phenomenon may be attributed to the complex mixture of impact damage in the woven plies and non-woven plies.

The accuracy of the fitted line is analyzed by calculating the corresponding damage size on the fitted line with the ToF delay after each impact in each plate. In woven CFRP plate, the calculated corresponding damage size is about 34% larger than the actually obtained damage size after the second impact, and is about 18% smaller after the fourth impact. In non-woven CFRP plate, the calculated damage size is 94% larger after the first impact and is 18% smaller after the fourth impact when compared with the actual size. As for the hybrid CFRP plate, the calculated damage size is about 29% larger and 29% smaller than the actual obtained damage size after the third and the fifth impact respectively. These results also suggest that the result for the woven CFRP has the best linearity.

### 4.3. Influence of Damage Shape on the ToF and the Amplitude of the A_0_ Mode

**Figure 12:**
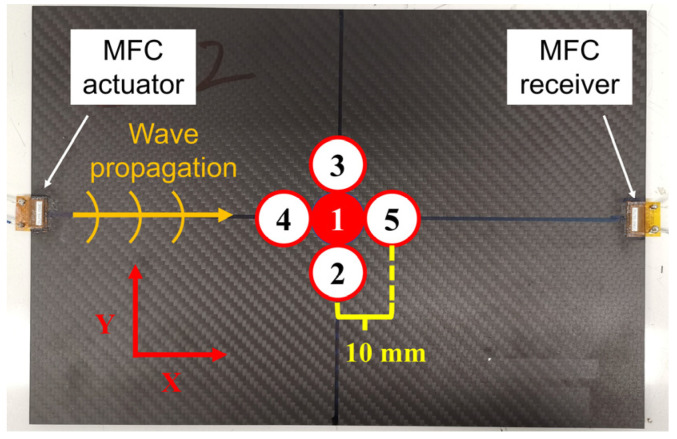
Positions of the five impacts in woven CFRP plate No. 2.

In Section 4.2, the shape of the impact damage in the woven CFRP plate changed after each impact loading. In this section, we describe an experiment that was conducted to investigate the influence of the impact damage shape on Lamb waves. The experimental setup was the same as that shown in Figure 2. Another woven CFRP plate ([(0, 90)/(45, −45)]_4s_, 3.64 mm in thickness) with the same layup configuration and size was used. The woven CFRP specimen used in Section 4.2 was designated as woven CFRP plate No. 1, and the specimen used in this experiment was designated as woven CFRP plate No. 2. In this experiment, in contrast to Section 4.2, the impact damage was first enlarged in the Y direction by the second and third impacts, and then in the X direction by the fourth and fifth impacts, as shown in Figure 12. 

**Figure 13:**
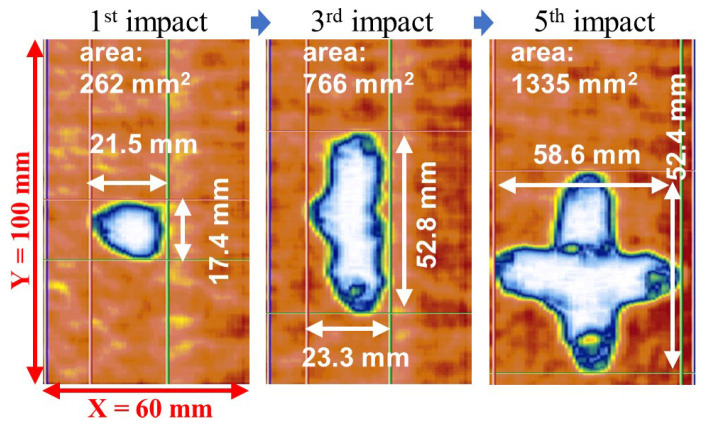
C-scan observation results of impact damages in woven CFRP plate No. 2.

Figure 13 shows the C-scan result for woven CFRP plate No. 2, which confirms that the impact damage is enlarged in the second and third impacts in the Y direction as expected, which is different from the result of woven CFRP plate No. 1 in Figure 8a. After the fifth impact, the shape of the impact damage became a four-point star, which is similar to the shape of the impact damage in woven CFRP plate No. 1. 

**Figure 14:**
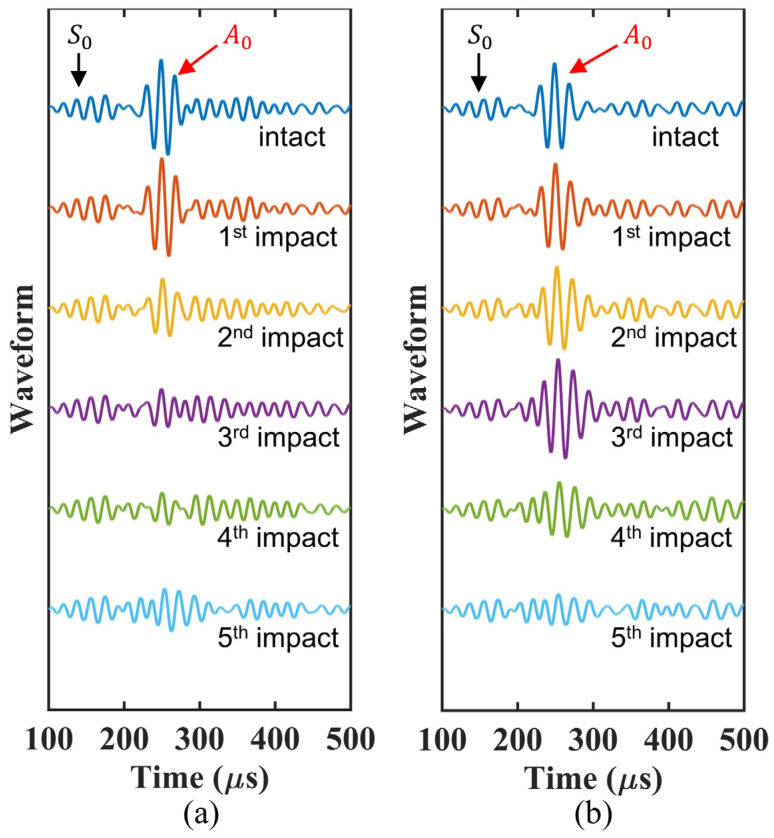
Reconstructed signals at 50 kHz in (**a**) woven CFRP plate No. 1 and (**b**) woven CFRP plate No. 2.

The same experiment of the chirp ultrasonic propagation was conducted for the woven CFRP plate No. 2. To determine other features in the waveforms that are sensitive to the impact damage shape, we extracted the reconstructed waveforms at 50 kHz. Note that the ToF delay has a maximum value of approximately 50 kHz. Figure 14 shows the changes in the waveforms that are caused by the change in the damage shape. The A_0_ mode can be clearly identified as marked. In woven CFRP plate No. 1, the amplitude of the A_0_ mode decreased significantly after the second and third impacts, whereas an obvious decrease was observed after the fourth and fifth impacts in woven CFRP plate No. 2.

**Figure 15:**
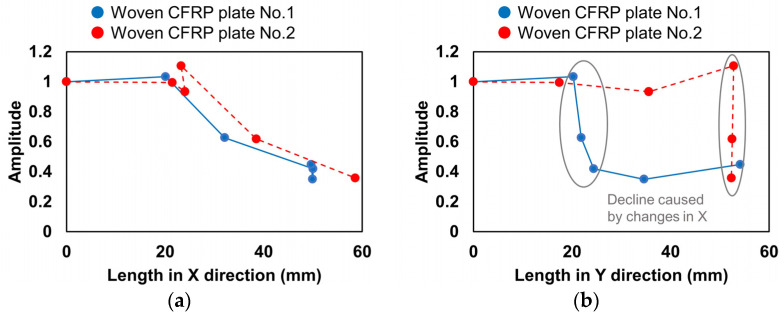
Relationship between the amplitude of the A_0_ mode and damage lengths in (**a**) X direction and (**b**) Y direction.

The amplitude of the A_0_ mode obtained from the above waveforms was plotted against the damage length in the X and Y directions, as shown in Figure 15. To compare the two plates, the amplitude of the A_0_ mode was normalized to the amplitude in the intact condition. The amplitude of the A_0_ mode decreased significantly as the impact damage increased in the X direction, while no obvious relationship was found between the amplitude of the A_0_ mode and the damage length in the Y direction. Thus, the amplitude of the A_0_ mode can be used to identify the extension direction of the impact damage as a complement to the ToF delay distribution.

## 5. Conclusions

In this study, the detection of impact damage by exciting and receiving broadband ultrasonic Lamb waves using MFCs was investigated to develop an SHM system in mobility structures made of CFRP. First, the cross-section of the impact-damaged area in each type of plate was observed, and the difference in the damage conditions of the three different CFRP laminates was clarified. Then, the relationship between the impact damage size and the maximum ToF delay was investigated for the three types of plates. As a result, the relation was found to be approximately linear for all the plates, despite the difference in microscopic damage. It was also found that the attenuation of the A_0_ mode was largely affected by the damage length in the wave propagation direction, which can be used to identify the extension direction of the impact damage as a complement to the ToF delay distribution. Thus, this SHM method using chirp ultrasonic waves can quantitatively evaluate the size and extension of the impact damage shape in mobility structures made of CFRP laminates.

In this study, impact damage occurred at the middle of the line between the two MFCs. However, in practical applications, impact damage may occur anywhere in the entire structure. The goal of our future research will be to detect the damage located outside the line between the two MFCs by observing scattering waves. This work will contribute to the development of a more reliable SHM method for CFRP mobility structures.

## Data Availability

Not applicable.
